# Whole-Genome Sequencing Analyses of Heat-Resistant Escherichia coli Isolated from Brazilian Beef

**DOI:** 10.1128/mra.00371-22

**Published:** 2022-07-21

**Authors:** Maxsueli Aparecida Moura Machado, Vinicius Silva Castro, Ricardo César Tavares Carvalho, Eduardo Eustáquio de Souza Figueiredo, Carlos Adam Conte-Junior

**Affiliations:** a Postgraduate Program in Food Science, Chemistry Institute, Federal University of Rio de Janeiro, Rio de Janeiro, Rio de Janeiro, Brazil; b Center for Food Analysis, Technological Development Support Laboratory, Federal University of Rio de Janeiro, Rio de Janeiro, Rio de Janeiro, Brazil; c Laboratory of Advanced Analysis in Biochemistry and Molecular Biology, Department of Biochemistry, Federal University of Rio de Janeiro, Rio de Janeiro, Rio de Janeiro, Brazil; d Postgraduate Program in Nutrition, Food and Metabolism, Federal University of Mato Grosso, Cuiabá, Mato Grosso, Brazil; e Postgraduate Program in Animal Science, Federal University of Mato Grosso, Cuiabá, Mato Grosso, Brazil; f University of Lethbridge, Lethbridge, Alberta, Canada; g University of Cuiabá, Animal Bioscience Postgraduate Program, Cuiabá, Mato Grosso, Brazil; University of Maryland School of Medicine

## Abstract

Four Escherichia coli isolates with moderate or high heat resistance were sequenced. Through sequencing, truncated transmissible locus of stress tolerance (tLST) variants tLST1 and tLSTa were identified in the three isolates. The most identified tLST genes (*clpK* and *hsp*) are responsible for the homeostasis module.

## ANNOUNCEMENT

Escherichia coli bacteria can be present in warm-blooded animals (1) and can exhibit a high heat resistance phenotype (2–4). This resistance has been related to tLST, previously termed the locus of heat resistance (LHR) (4, 5). Therefore, we evaluated 22 E. coli strains isolated from beef in Brazilian slaughterhouses. The strains were confirmed by biochemical tests and PCR by Castro et al. and Santos et al. (6, 7). Additionally, these strains were heat treated (60°C for 0 or 6 min) in a water bath. Four of the strains showed high (reduction of <1 log CFU/mL) or moderate (reduction of 1 to 5 log CFU/mL) resistance (4) (Table 1). Three strains were positive for the presence of tLST, through PCR (8). The positive strains were properly stored at −80°C in brain heart infusion (BHI) broth with glycerol and were streaked on MacConkey agar, with subsequent incubation at 37°C for 18 to 24 h. After that, a characteristic colony was inoculated on BHI broth and incubated at 37°C for 18 to 24 h. Subsequently, these cultures were used for DNA extraction using the DNeasy blood and tissue kit (Qiagen, Hilden, Germany). The DNA concentration was assessed using a fluorescence technique (Qubit 2.0 system; Invitrogen, Grand Island, NY, USA). Thereafter, whole-genome sequencing was performed using the NovaSeq 6000 platform, with paired-end reads (2 × 150 bp) (Illumina Inc., San Diego, CA, USA). Sample libraries were prepared using the NEBNext Ultra II DNA kit (New England Biolabs, Ipswich, MA, USA). The genome assembly used Shovill with SPAdes v.1.1.0 as assembler (https://github.com/tseemann/shovill), with trim reads enabled (Trimmomatic v.0.38). The sequencing quality report was accessed using QUAST v.5.0.2 and FastQC v.0.11.8, with default parameters applied for both. The genomic annotation was obtained using PGAP v.pgap-5.3 (update 2021-11-29.build5742) for GenBank submission, with default parameters applied. For tLST investigation, an initial search with BLAST v.2.13.0 was performed using our sequences versus tLST variants (tLST1 [GenBank accession number LDYJ01000141], tLSTa [GenBank accession number CP010237], tLST2_C604-10_ [GenBank accession number CP016838], and tLST2_FAM21805_ [GenBank accession number KY416992]) described by Wang et al. (9). For this search, default parameters are applied, such as an expected threshold of 0.05 and match and mismatch scores of 1, -2. The matches of the tLST sequences characterized by Wang et al. (9) and our isolates showed 42, 42, and 43% nucleotide identity for strains C31, C1145, and C09, respectively, for the tLST1 variant. Strain C97 showed only 12% similarity to the tLSTa variant using BLAST analysis.

**TABLE 1:** Characteristics of genome assemblies from E. coli isolates

Strain	Heat resistance phenotype^*a*^	BioSample accession no.	SRA accession no.	Assembly accession no.	Total no. of reads	Coverage (×)	G+C content (%)	*N*_50_ (bp)	No. of contigs	Genome size (bp)	tLST genes detected^*b*^
C97	Moderate	SAMN25948547	SRR18038022	ASM2235984v1	10,945,256	31	50	325,369	156	4,856,118	*HdeD*, *degP*
C09	High	SAMN25948544	SRR18038025	ASM2235989v1	7,786,050	28	50	86,054	305	5,278,357	*hspA_1*, *hspA_2*, *clpK*, *yfdX1*, *yfdX2*, *degP*, *HdeD*
C1145	High	SAMN25948546	SRR18038023	ASM2235987v1	17,704,384	36	51	150,778	175	4,953,542	*hspA_1*, *hspA_2*, *clpK*, *degP*, *HdeD*
C31	High	SAMN25948545	SRR18038024	ASM2240563v1	13,126,568	38	51	150,778	166	4,949,474	*hspA_1*, *hspA_2*, *clpK*, *degP*, *HdeD*

a Strains classified as moderate had reductions of 1 to 5 log units and those classified as high showed reductions of <1 log unit after treatment at 60°C for 6 min in a water bath, following criteria established by Mercer et al. (4).

b Genes were identified using tLST-annotated assemblies as a database in Geneious Prime v.2022.0.2. This database was used to annotate our sequences with a degree of similarity of 90%, allowing truncated genes.

After determination of the tLST range by BLAST analysis when high linkage identity was present (>90%), a database containing tLST-annotated assemblies (GenBank accession numbers ASM130945v1, ASM190098v1, ASM196942v1, and KY416992.1) was created in Geneious Prime v.2022.0.2. The analysis consisted of annotating our sequences using tLST genome assemblies (9). The best matches between the genes present in the tLST sequences and our genomes were used with a degree of similarity of 90%, allowing the annotation of truncated genes. Some tLST genes, as well as genome coverage information, total sequence lengths, *N*_50_ values, and G+C contents, are detailed in Table 1.

### Data availability.

The BioProject accession number for the raw sequence reads is PRJNA806981.
